# Quantitative Evaluation of the Transcriptional Activity of Steroid Hormone Receptor Mutants and Variants Using a Single Vector With Two Reporters and a Receptor Expression Cassette

**DOI:** 10.3389/fendo.2020.00167

**Published:** 2020-03-31

**Authors:** Huimin Ji, Ying Li, Zhao Liu, Min Tang, Lihui Zou, Fei Su, Yaqun Zhang, Junhua Zhang, Hexin Li, Lin Li, Bin Ai, Jie Ma, Lunan Wang, Ming Liu, Fei Xiao

**Affiliations:** ^1^National Center for Clinical Laboratories, Beijing Hospital, National Center of Gerontology, Institute of Geriatric Medicine, Chinese Academy of Medical Sciences, Beijing, China; ^2^Beijing Engineering Research Center of Laboratory Medicine, Beijing Hospital, Beijing, China; ^3^Graduate School, Peking Union Medical College, Chinese Academy of Medical Sciences, Beijing, China; ^4^The Key Laboratory of Geriatrics, Beijing Institute of Geriatrics, Beijing Hospital, National Center of Gerontology, National Health Commissions, Institute of Geriatric Medicine, Chinese Academy of Medical Sciences, Beijing, China; ^5^Department of Thyroid and Breast Surgery, The Affiliated Hospital of Xuzhou Medical University, Xuzhou, China; ^6^Department of Oncology, Beijing Hospital, National Center of Gerontology, Institute of Geriatric Medicine, Chinese Academy of Medical Sciences, Beijing, China; ^7^Clinical Biobank, Beijing Hospital, National Center of Gerontology, Institute of Geriatric Medicine, Chinese Academy of Medical Sciences, Beijing, China; ^8^Department of Urology, Beijing Hospital, National Center of Gerontology, Institute of Geriatric Medicine, Chinese Academy of Medical Sciences, Beijing, China; ^9^Center for Biotherapy, Beijing Hospital, National Center of Gerontology, Institute of Geriatric Medicine, Chinese Academy of Medical Sciences, Beijing, China; ^10^State Key Lab of Molecular Oncology, National Cancer Center, Chinese Academy of Medical Sciences and Peking Union Medical College, Beijing, China; ^11^Department of Pathology, Beijing Hospital, National Center of Gerontology, Institute of Geriatric Medicine, Chinese Academy of Medical Sciences, Beijing, China

**Keywords:** steroid hormone receptors (SHRs), mutations, splicing variations, dual-luciferase reporter assay, transactivation activity

## Abstract

Although the rapid development of high-throughput sequencing has led to the identification of a large number of truncated or mutated steroid hormone receptor (SHR) variants, their clinical relevance remains to be defined. A platform for functional analysis of these SHR variants in cells would be instrumental for better assessing their impact on normal physiology and SHR-associated diseases. Here we have developed a new reporter system that allows rapid and accurate assessment of the transcriptional activity of SHR variants in cells. The reporter is a single construct containing a firefly luciferase reporter gene, whose expression is under the control of a promoter with multiple steroid hormone responsive elements, and a *Renilla* luciferase reporter gene, that is constitutively expressed under the control of an internal ribosome entry site (IRES) and is not regulated by steroid hormones. The corresponding SHR (wildtype or mutant/variant) is also expressed from the same construct. Using this improved reporter system, we revealed a large spectrum of transactivation activities within a set of previously identified mutations and variations of the androgen receptor (AR), the estrogen receptor α (ERα) and the glucocorticoid receptor (GR). This novel reporter system enables functional analysis of SHR mutants and variants in physiological and pathological settings, offering valuable preclinical, or diagnostic information for the understanding and treatment of associated diseases.

## Introduction

Many SHR variants have been identified in both common and rare diseases (1–3). All SHRs share a similar domain organization, including a C-terminal ligand-binding domain (LBD), a hinge region, a DNA-binding domain (DBD), and a N-terminal domain (NTD). SHR variants may have cis- or trans-effects on multiple genes. Naturally existing GR variants (truncated or mutated receptors) for example, are one of the main causes of corticosteroid resistance (1), and mutations in AR could cause androgen insensitivity syndrome (AIS), spinal and bulbar muscular atrophy, and prostate cancer (2). Alterations in SHRs are also a common underlying mechanism for drug resistance in cancer therapy (4). For instance, splicing variations and mutations in AR are associated with the progression of castration-resistant prostate cancer (CRPC) (5). Mutations in ERα have also been identified in hormone therapy resistant breast cancer treated with selective ERα modulators such as tamoxifen and raloxifene (3). Although many disease-associated SHR splicing variants and mutants have been identified, very few of them have been functionally analyzed, in part, due to the lack of a general platform that can quantitatively evaluate and compare the transcriptional activities of SHR variants and mutants.

Luciferase reporter assays have been used for studying many cellular processes including intracellular signaling, gene expression, receptor activity, transcription factors, mRNA processing, and protein folding. Dual luciferase reporter systems have been used in the past to assay SHR activity in gene regulation with improved accuracy by using one luciferase as an internal control. These systems usually involve co-transfection of three expression vectors (including the SHR expression vector, the experimental reporter gene vector, and the internal control reporter gene vector) into a cell line, which could introduce additional variables and thus impact assay accuracy. In this study we engineered multiple single-vectored, dual-luciferase reporters that can rapidly and accurately measure the activity of individual SHRs in cells. These reporters contain a constitutively expressed *Renilla* luciferase reporter gene under the control of an internal ribosome entry site (IRES), an SHR-expressing cassette, and a firefly luciferase reporter gene driven by a promoter that is regulated by the corresponding SHR. Using these improved reporter systems, we undertook a comprehensive survey of a large number of AR, ERα and GR variants that were previously identified in clinical or preclinical studies. Our results reveal distinct transcriptional activities of these variants, providing insights into their roles in the pathogenesis of associated diseases.

## Materials and Methods

### Cell Lines and Culture Conditions

The Huh-7 and COS-7 cells were obtained from Peking Union Medical College (PUMC, Beijing, China). PC3 cells were cultured in RPMI-1640 medium (Gibco) supplemented with 10% FBS (Gibco) in a humidified atmosphere with 5% CO_2_. Huh-7, COS-7, Hela, HEK293T, and HepG2 cells were cultured in phenol DMEM(H) medium (Thermo Fisher Scientific, Waltham, MA USA) supplemented with 10% FBS (Gibco) at 37°C in a humidified atmosphere with 5% CO_2_.

### Cloning of Constructs

PSA61-Luc(obtained from Jan Trapman and Hetty van der Korput, Erasmus MC, Netherlands), pcDNA3.1(+)-AR and pSG5-hERα were kindly provided by Jörg Klug, JLU Giessen, Germany. 4×ARE-Luc and 2×GRE-Luc plasmids were kindly provided by Prof. Michael Carey at UCLA and Iain J. McEwan at University of Aberdeen, respectively. The ARE-I-II-III-Luc vector was generated by replacing the PSA61 region in PSA61-Luc (between the BamHI and EcoRI sites) with an ARE-I-II-III fragment (amplified through overlapping PCR).

The pcDNA3.1(-)-4×ARE-Fluc-AR-Rluc was generated through the following steps: (a) the CMV promoter in pcDNA3.1(-) vector was replaced with the 4×ARE and minimal promoter, amplified from the 4×ARE-Luc plasmid; (b) the firefly luciferase gene was inserted into the pcDNA3.1(-)-4xARE vector downstream of the minimal promoter; (c) the neomycin gene in the pcDNA3.1(-)-4×ARE-Fluc was replaced with IRES and the *Renilla* luciferase reporter gene; (d) the full length wild type AR fragment was then inserted upstream of the IRES sequence in pcDNA3.1(-)-4×ARE-Fluc-Rluc between the XhoI and XbaI sites.

To generate pcDNA3.1-4×ARE-Fluc-AR-Vs-Rluc constructs, various AR variant sequences (AR-Vs) were amplified by PCR from pcDNA3.1(+)-AR based on previous studies (6–10), which were then used to replace the AR region (between the XhoI and XbaI sites) in pcDNA3.1-4×ARE-Fluc-AR-Rluc.

To generate pcDNA3.1-4×ERE-Fluc-ERα-Vs-Rluc plasmids, a 4xERE promoter sequence containing four copies of ERE was synthesized by Qingke Biotech (Beijing, China) a. ERα wildtype cDNA was amplified from pSG-hERα and used to replace the AR cDNA in pcDNA3.1(-)-4×ARE-Fluc-AR-Rluc. ERα variant and mutant sequences were generated by overlapping PCR and used to replace ERα-wt cDNA in the vector to generate pcDNA3.1-4×ERE-Fluc-ERα-Vs-Rluc expression constructs.

To generate pcDNA3.1(-)-4×GRE-Fluc-GR-Vs-Rluc, the 4xGRE promoter sequence was amplified from the 2×GRE-Luc vector by overlapping PCR, and the wildtype GR cDNA was amplified from cDNA isolated from HeLa cell, as described (11). 4×GRE and wildtype GRcDNA were then used to replace 4×ARE and the AR-wt cDNA in pcDNA3.1(-)-4×ARE-Fluc-AR-Rluc. GR-wt cDNA was then replaced with different GR variants cDNA generated by overlapping PCR.

Sequences of all the primers used to create AR-wt, ERα-wt, and GR-wt cDNA, as well as their corresponding variants and mutations, are described in Tables S1–S6. The sequences of ARE, GRE and ERE are provided in the Table S7.

### Luciferase Reporter Assays

HEK293T cells were seeded in 6-well plates and cultured in complete medium containing 10% charcoal-stripped fetal bovine serum (Sijiqing, ZhejiangTianhang, Biotechnology, China) for 24h. Subsequently, they were transfected with different constructs using the Lipofectamine 3000 transfection reagent (ThermoFischer Scientific Inc., Waltham, MA, USA) according to the manufacturer's protocol. Six hours after transfection, the medium was replaced with fresh charcoal-stripped medium containing either corresponding hormones [R1881 (Yuanye, Shanghai, China), diethylstilbestrol (Aladdin, Shanghai, China), dexamethasone (Sigma-Aldrich, Sweden)], or DMSO solvent (Sangon, Shanghai, China). After 48 h of incubation, cells were then washed, lysed, and harvested. Dual-Luciferase Reporter Assay (Promega, Madison, WI) was then performed in duplicates to measure the activities of firefly luciferase and *Renilla* luciferase. Transfection efficiency was normalized by *Renilla* luminescence signals. The results are presented as the mean ± SD of triplicate samples.

### Statistical Analysis

All reported values represent the average of triplicates. Statistical analysis of differences between samples was performed using two-tailed student's *t*-test and *P* < 0.05 is considered significant. Data are presented as mean ± SD. Analysis was conducted using Prism v6.0c (GraphPad Software).

## Results

### Selection for Appropriate Transfected Cell Lines, Effective Promoters, and Optimal Stimulus Concentration

In general, activation of receptors requires not only ligands binding but also recruitment of various coactivators to the transcriptional complex. Thus, an appropriate cellular context and a sensitive and effective promoter are important factors for consideration for assessing AR transcriptional activity. After surveying androgen regulation of PSA61-Luc in six AR-negative cell lines, we found the promoter displayed the highest fold stimulation activity (16.6-fold change) in HEK293T compared to other cell lines (Figure S1). Whereas ARE-I-II-III and 4×ARE reporters displayed 11.6- and 55-fold change stimulation in HEK293T (Figure S2). Therefore, we chose HEK293T and 4×ARE-Luc to examine the transcriptional activity of AR-wt.

**Figure 1:**
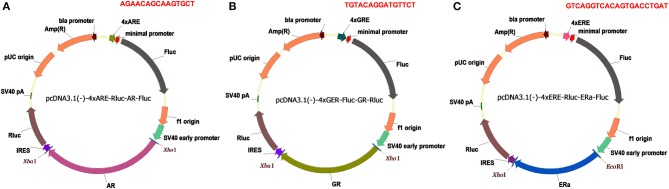
Dual luciferase reporters for SHRs. **(A)** pcDNA3.1(-)-4xARE-Fluc-AR-Rluc reporter. **(B)** pcDNA3.1-4xGRE-Fluc-GR-Rluc reporter. **(C)** pcDNA3.1-4xERE-Fluc-ERa-Rluc reporter. Amp(R), ampicillin resistance gene; Fluc, firefly luciferase gene; Rluc, *Renilla* luciferase gene; SV40 early promoter, simian vacuolating virus 40 early promoter; IRES, internal ribosome entry site; 4×ARE, four copies of androgen responsive elements; 4×ERE, four copies of estrogen responsive elements; 4×GRE, four copies of glucocorticoid responsive element; the responsive element sequences are highlighted in red.

In order to assess the effects of AR alterations in low androgen concentrations in castration environments, we constructed pcDNA3.1-4×ARE-Fluc-AR-wt-Rluc reporter (Figure 1A) and transfected it into HEK293T. As shown in the Figure S3A, 10nM R1881, which confers comparable stimulation effects with 100nM R1881 but higher stimulatory effects than 0.1 or 1 nM R1881, is chosen for comparing the transcriptional activity of AR-wt and its mutants or variants. Similarly, we constructed pcDNA3.1-4×ERE-Fluc-ERα-wt-Rluc and pcDNA3.1-4×GRE-Fluc-GR-wt-Rluc reporters (Figures 1B,C) and chose 1.0nM diethylstilbestrol (DES) and 10nM dexamethasone (DEX) physiologically-relevant concentration as the ligand concentration to evaluate the transcription activity of ERα variants and GR variants, respectively (Figures S3B,C).

### Effects of Prostate Cancer-Associated AR Mutations and Splicing Variations on Transcription

After surveying six different cell lines and three promoters displaying different efficiency of activation, we chose HEK293T and 4×ARE-Luc to compare the transcriptional activity of AR-wt and its mutants or variants (Figures S1, S2A,B). In order to assess the effects of AR alterations in low androgen concentrations in castration environments, we chose the 10 nM R1881 physiological concentration (Figure S3A).

**Figure 2:**
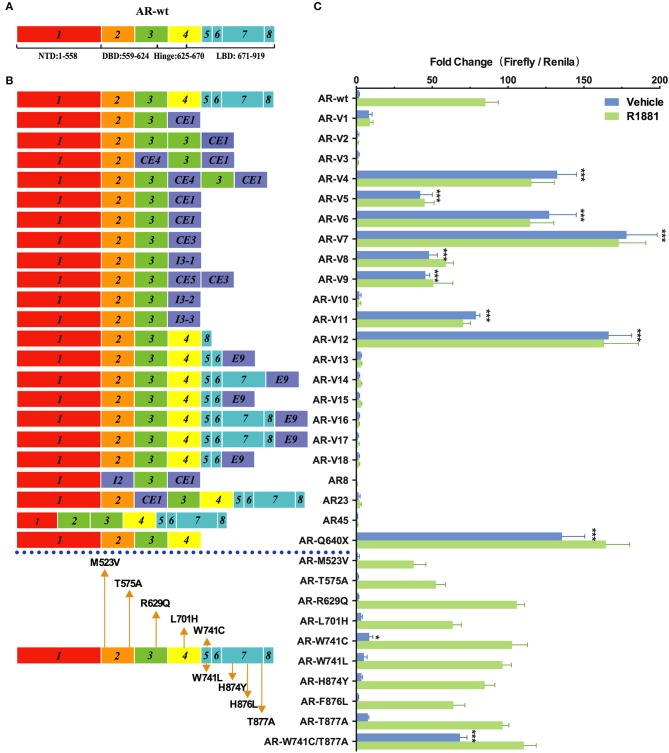
Effects of AR mutations and splicing variations on transcription. **(A)** Domain structure of AR-wt. **(B)** AR mutants and variants tested. **(C)** Transcriptional activity of transfected AR mutants in HEK293T cells treated with 10 nM of R1881 or vehicle for 48 h. Results represent the mean ± SD of three biological replicates. *P <0.05; ***P <0.001. AR, androgen receptor; AR-FL, full-length androgen receptor; AR-Vs, androgen receptor variants; AF, activation function; CE, cryptic exon; CRPC, castration-resistant prostate cancer; DBD, DNA-binding domain; I, intron; LBD, ligand-binding domain; NTD, N-terminal domain; NLS: nuclear localization sequence.

As shown in Figure 2, nine variants (AR-V4, AR-V5, AR-V6, AR-V7, AR-V8, AR-V9, AR-V11, AR-V12, and ARQ640X) exhibited constitutive transcriptional activity in the absence of R1881. Consistent with the previous reports (6, 7), AR-V7 and AR-V12 (also known as AR v567es), both of which are the most frequently expressed variants in clinical specimens, displayed the highest luciferase activity in the absence of R1881 (which were 91 and 81 more active than AR-wt, respectively) (Table 1). Q640X, a truncation mutant with a point mutation that leads to a premature stop codon at position 640 of the AR, strongly activated 4×ARE promoter (71 times of the basal activity of AR-wt), in agreement with a previous observation (23). AR-V7 and ARV567es exhibited similar transcriptional activity to Q640X (Figure 2). Less-studied AR-Vs such as AR-V4, AR- V5 and AR-V6 were found in CRPC metastases, with lower expression levels than AR-V7 or AR-V9(13). However, low-abundance AR-Vs including AR-V4, AR-V5, AR-V6, AR-V8 and AR-V11 displayed remarkable constitutive activity in stimulating transcription (69×, 22×, 66×, 25×, 41×, and 25×basal activity of AR-wt, respectively). AR-V9 displayed modest transcriptional activity (24×basal activity of AR-wt) in a ligand-independent manner, in line with previous observations (12). Because some variants such as AR-V7 and AR-V9 are frequently co-expressed in CRPC metastases (12, 13), and because AR-Vs can form heterodimers/homodimers (24), it is possible that their combined contribution to prostate cancer progression might be greater than their individual effects.

**Table 1:** Selected resistance mechanisms by type of androgen receptor (AR) or estrogen receptor modification.

**Modification**	**Clinical relevance**	**Biological relevance**	**Mechanism**	**References**
**AR variants**				
AR-V7	Enriched in CRPC and metastasis, high risk of recurrence, decreased PFS and OS of CRPC patients	Resistant to ADT, enzalutamide and abiraterone	Constitutive activation	(6)
AR-V9	Enriched in CRPC and metastasis decreased PFS of CRPC patients	Resistant to ADT, enzalutamide and abiraterone	Conditional activation	(12)
AR-V12 (ARv567es)	Enriched in CRPC and metastasis decreased PFS of CRPC patients	Resistant to ADT, drives invasive adenocarcinoma *in vivo*	Constitutive activation	(7)
**AR mutations**				
L701H	Existed in metastatic CRPC	Activated by glucocorticoids	Receptor promiscuity	(13)
W741C	Existed in metastatic CRPC	Activated by bicalutamide, flutamide	Antagonist-to-agonist switch	(5)
W741L	Existed in metastatic CRPC	Resistant to Abiraterone	Antagonist-to-agonist switch	(14)
H874Y	Existed in metastatic CRPC	Activated by estrogen, progesterone, glucocorticoids, adrenal androgens, bicalutamide, flutamide, enzalutamide and apalutamide	Antagonist-to-agonist switch, Receptor promiscuity	(15)
F876L	Existed in metastatic CRPC	Activated by flutamide, apalutamide and enzalutamide	Antagonist-to-agonist switch	(16)
T877A	Existed in metastatic CRPC	Activated by progesterone, estrogen, flutamide, bicalutamide, enzalutamide and apalutamide	Antagonist-to-agonist switch, Receptor promiscuity	(5, 17)
**ER mutations**				
Y537S	Existed in metastatic ER+ breast cancer, decreased PFS and OS	Resistance to the beneficial effects of the SERM, SERD, and AIs.	Constitutive activation	(18–20)
D538G	Existed in metastatic ER+ breast cancer, decreased PFS and OS	Resistance to the beneficial effects of the SERM, SERD, and AIs.	Constitutive activation	(18–20)
**GR mutations**				
D641V	Existed in generalized glucocorticoid resistance	Reduced sensitivity to glucocorticoids	Decreased bind ability to GRE; abnormal interaction with GRIP1; dominant negative effect on GR-wt	(11)
R477H	Existed in Hirsutism and generalized glucocorticoid resistance	Reduced sensitivity to glucocorticoids	Decreased affinity to GREs	(21)
V729I	Existed in generalized glucocorticoid resistance with homosexual precocious puberty	Reduced sensitivity to glucocorticoids	Decreased interaction with NCoA, affinity to ligand and nuclear translocation;hamper the formation of homodimer	(22)

*CRPC, castration resistant prostate cancer; ADT, androgen deprivation therapy; PFS, progression-free survival; OS, overall survival; AIs, aromatase inhibitors; SERM, selective estrogen receptor modulators; SERD, selective estrogen receptor degraders; GRE, glucocorticoid responsive elements; GRIP1, recombinant glutamate receptor interacting protein 1; NCoA, nuclear receptor coactivators*.

Among the 22 AR variants examined, 13 displayed a complete loss of function in the presence or in the absence of R1881. Some of them, including AR-V1, AR-V2, AR23, AR45, and AR8, have been characterized before (6, 9, 25, 26). They were previously suggested to function through a non-genomic mechanism or cytoplasmic action (9, 25). However, other variants (e.g., AR-V10, AR-V13, AR-V14, AR-V15, AR-V16, AR-V17 and AR-V18) were identified more recently in certain cell lines or tissues and have not been functionally analyzed (8, 27). AR-V3, which was exclusively found in CRPC (13), was previously suggested to display constitutive transcriptional activity that is even higher than the reported activity of AR-V7 in DU-145 cells (28). However, in our analysis, this variant was completely inactive.

AR mutations are rare in the early stages of untreated PC in patients, but occur much more frequently in castration-resistant prostate cancer (CRPC) (29). Gain-of-function mutations in AR allow prostatic epithelial cells to grow in an androgen-independent manner. In our study, the double mutant W741C/T877A exhibited higher transcription activity (36×basal AR-wt levels) than W741C or T877A single mutant in the absence of hormone, and this activity was further enhanced by R1881 (Figure 2). This indicates that W741C and T877A mutations may cooperate to confer new properties on AR. L701H, W741L/C, H874Y, and F876L, all of which were detected in CRPC patients (Table 1), displayed similar transcriptional activity to AR-wt. The R629Q mutation in the hinge region important for DNA binding and nuclear translocation of AR, caused a mild increase of transcriptional activity in the presence of R1881 (1.3×basal AR-wt levels) (Figure 2). M523V and T575A, both of which were detected in metastatic PCa and patients receiving combined androgen blockade, displayed a modest decease of transcriptional activity (44 and 62% of AR-wt levels) in the presence of R1881 (Figure 2).

### Effects of Disease-Associated ERα Mutations and Splicing Variations on Transcription

**Figure 3:**
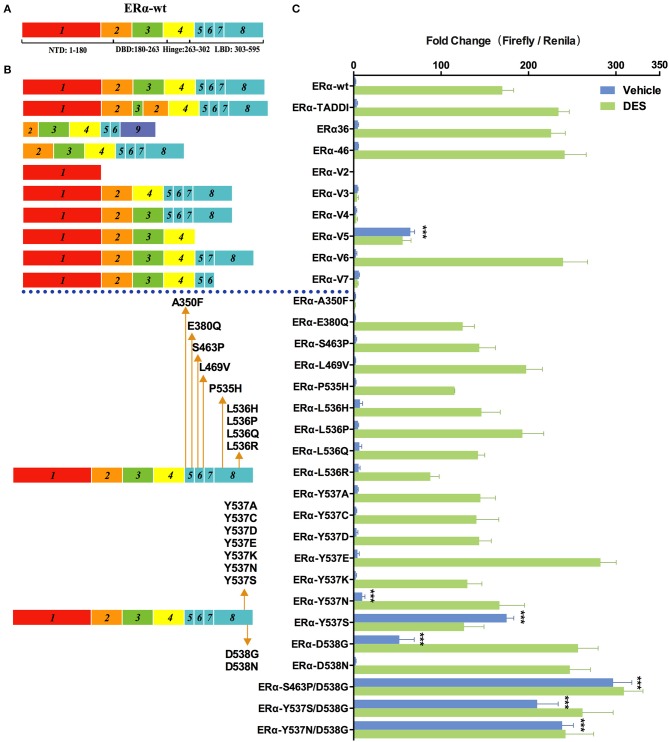
Effects of ER mutations and splicing variations on transcription. **(A)** Domain structure of ERα-wt. **(B)** Location of tested mutations in ERα. **(C)** Transcriptional activity of transfected ERα mutants in HEK293T cells treated with 1 nM of diethylstilbestrol (DES), or vehicle for 48 h. Results represent the mean ± SD of three biological replicates. ^***^P <0.001. ERα-wt, wide-type estrogen receptor-α; ERα-Vs, estrogen receptor variants; DBD, DNA-binding domain; LBD, ligand-binding domain; NTD, N-terminal domain.

Since discovered in early 1990s, many naturally occurring splice variants of ERα with different coding sequences have been identified in cell lines and clinical samples (30, 31). To examine the transcriptional activity of these ERα variants, we constructed a series of pcDNA3.1-4×ERE-Fluc-ERα-Rluc reporters expressing WT and ERα variants (Figure 1B). We chose 1.0 nM diethylstilbestrol (DES), which confers maximal stimulatory effects on the 4×ERE promoter (Figure S3B), as the ligand concentration to evaluate the transcription activity of ERα variants. Among the nine ERα splice variants examined, only ERα-V5 (which lacks the exon 5) exhibited modest transcriptional activity (25×basal ERα-wt levels) in the absence of DES (which is consistent with previous reports) (Figure 3) (31, 32). The TADDI variant, which contains a 31bp deletion between exons 3 and 4 and a 13 bp insertion in this splice site and which is increased in postmenopausal women in the hippocampus, is 1.42 times more active than ERα-wt in stimulating transcription in the presence of DES (Figure 3). The ERα-V6, ERα36, and ERα46 variants displayed a similar transcriptional activity (1.4×basal ERα-wt levels) in the presence of DES (Figure 3). The other four variants (ERα-V2, ERα-V3, ERα-V4, and ERα-V7) apparently have no transcriptional activity (Figure 3), consistent with previous reports (30, 33).

In addition to splicing variants, we also examined the transcriptional activity of ERα mutants which have been identified in 10 to 54.5% metastatic, ERα-positive breast cancers (18, 19, 34). Among the 20 cancer-relevant mutants examined, S463P/D538G showed the highest levels of transcriptional activity in the absence of DES (133×basal ERα-wt levels), which is followed by Y537N/D538G and Y537S/D538G (108×and 95×basal ERα-wt levels), and single mutant Y537S, D538G, and Y537N (79×, 24×, and 5×basal ERα-wt levels). The above mutants, except for D538G and Y537N, induced a similar transcriptional activity in the presence of 1 nM of DES compared with themselves in the absence of hormone. In the presence of DES, D538G, and Y537N were both highly active and induced transcription to a similar level to that of Y537S/D538G. The ERα mutations described above occur frequently in various breast cancer samples, and only some of them have been characterized (18, 19) (Table 1). Patients with certain ERα mutations have a shorter progression-free survival (PFS) and overall survival (OS) following endocrine treatment, compared to patients with ERα-wt (20, 35, 36). In contrast to our results, Spoerke et al. did not find differential PFS in patients with ERα mutations compared with wild-type patients (37). However, that study included some other mutations including E380Q, S463P, Y536P, and Y537C, whose transcriptional ability tested in our system were similar to that of ERα-wt or even less sensitive than that of ERα-wt in the presence of DES. Thus, we hypothesize that not all mutations are related with the clinical outcomes, but mutations showing constitutive ability should be carefully focused on. Some mutations identified in advanced ER+ breast cancer exhibited similar (L469V, L536H, and L536Q) or a modest decease (P535H and L536R) of luciferase activity relative to ERα-wt in our study. Notably, A350F completely lost the transactivation activity in our study. These results suggest that the contribution of the transcriptional activity of ERα mutations to tumorigenesis is likely to be very complex.

Previous structural studies indicated that Y537S and D538G mutations cause increased interaction of ERα with its coactivators such as SRC-1, due to the shift of helix 12 to a conformation similar to that of estrogen-bound ERα-wt (38). Other mutations at the same site 537 and 538, including Y537A, Y537C, Y537D, Y537E, Y537K, and D538N, did not induce transactivation in the absence of DES, indicating the nature of the mutations is critical for the changes in ERα activity. Y537E and D538N exhibited 1.65- and 1.45-fold increase in transactivation activity, respectively, compared with ERα-wt, but only in the presence of DES.

### Effects of Disease-Associated GR Mutations and Splicing Variations on Transcription

**Figure 4:**
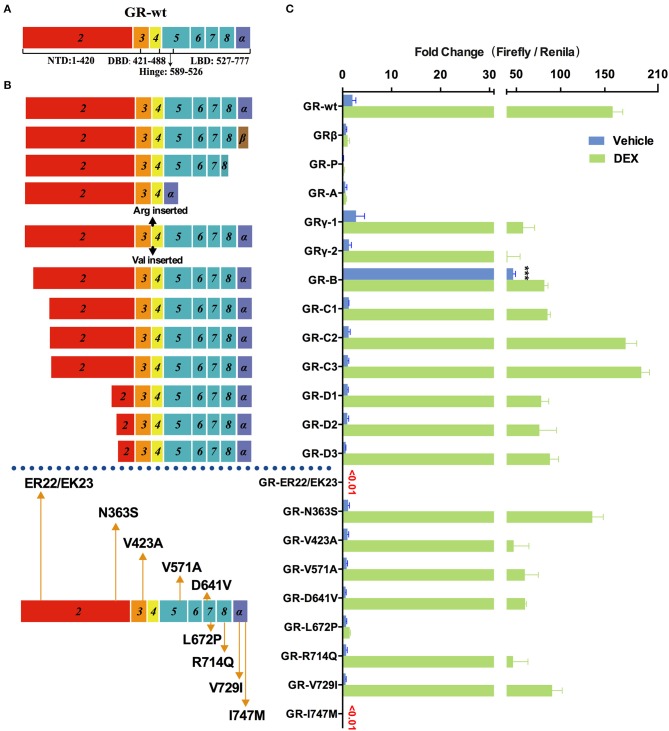
Effects of GR mutations and splicing variations on transcription. **(A)** Domain structure of GR-wt. **(B)** Location of tested mutations in GR. **(C)** Transcriptional activity of transfected GR mutants in HEK293T cells treated with 10 nM of dexamethasone(DEX) or vehicle for 48 h. Results represent the mean ± SD of three biological replicates. ^***^P <0.001. GR-wt, wide-type glucocorticoid receptor; GR-Vs, glucocorticoid receptor variants; DBD, DNA-binding domain; LBD, ligand-binding domain; NTD, N-terminal domain.

GR is another important member of nuclear receptor family that also contains multiple variants and is frequently mutated in cancer and other diseases. To investigate their transcriptional activity, we generated pcDNA3.1(-)-4×GRE-Fluc-GR-Rluc reporters expressing a serial of GR mutants and splice variants (Figure 4). The 10 nM DEX physiologically-relevant concentration was chosen for the comparisons of the transcriptional activity (Figure S3C).

Increased expression of GRβ (which does not contain helix 12 of LBD but has a different amino acid sequence in helix 11 compared to GR-wt) was observed in hormone-insensitive autoimmune diseases (39), blood system diseases (40), cancer (41), etc. As shown in Figure 4 transfected GRβ exhibited little transactivation activity in the presence or absence of glucocorticoids, consistent with previous studies (39, 40). When GR-wt and GRβ were co-transfected in a 1:1 ratio, the transactivation activity of GR-wt decreased dramatically. These observations raise the possibility that abnormally increased GRβ may decrease hormone sensitivity by inhibiting GR-wt.

The mRNA of GR-P variant accounts for 10–20% of total GR RNA in normal cells, which is much higher than GR-β. Certain blood cancer (e.g., multiple myeloma) and solid tumors (e.g., breast cancer) have increased GR-P, up to 50% of total GR-mRNA. However, it exhibited virtually no transactivation activity in our assay.

GR-A, which lost exons 5–7 during alternative splicing, lacks trans-acting regions and nuclear localization regions. Consistently, the observed transactivation activity of GR-A was minimal. The increase of GR-A expression may contribute to glucocorticoid insensitivity.

GR-γ, which is associated with hormone insensitivity in leukemia and non-small cell lung cancer (42), contains an arginine insertion between exons 3 and 4. The transactivation ability of GR-γ was significantly lower than that of GR-wt, although it was higher than GR-A, GR-P, and GRβ. Increased expression of GR-γ may also contribute to glucocorticoid insensitivity.

In addition to splicing variants, we also examined the transcriptional activity of multiple translation variants of GR. Expression of these translational variants is tissue- and cell- specific and might be one of the mechanisms underlying their different varying sensitivity. These translational variants might also contribute to glucocorticoid-related side effects. For instance, higher levels of translational variants C2 and C3 in some patients may result in higher glucocorticoids sensitivity and more frequent side effects such as glucocorticoid-induced osteoporosis. GR-C1, GR-D1, GR-D2, and GR-D3 exhibited clear loss of function compared with GR-wt in the presence of DEX. Both GR-C2 and GR-C3 had slightly higher transcriptional transactivation than GR-wt when treated with 10 nM DEX, consistent with a previous report (43). The transactivation activity of GR-B increased by several times compared with GR-wt in the absence of hormones (Figure 4), suggesting that GR-B might be associated with abnormally high GR activity under normal physiological conditions.

Primary glucocorticoid resistance due to mutations in the GR gene was found in 23 cases, most of which were single nucleotide mutations (21, 44), insertions (45), and deletions (46). Mutations occurred mostly in the ligand binding region, and have a significant effect on the function of GR. LP72P was first identified in a male patient who had no symptoms except bilateral adrenal hyperplasia, but function experiments showed that the mutant L672P had no transactivation ability (21). We also obtained a similar result (Figure 4). The I747M mutation was reported a family with primary glucocorticoid resistance as well as a series of other symptoms including irregular menstrual cycles, hyperandrogenism, cystic acne, and hairiness (47). Our result show that I747M also has extremely low transactivation ability.

We also explored the relationship between two common SNPs and glucocorticoid sensitivity. N363S had been reported to be associated with increased glucocorticoid sensitivity, decreased bone density (48), and frequent hormone-related side effects (49). In our study, however, the transactivation activity of N363S was actually slightly lower than that of the wild type, although the difference was not statistically significant (Figure 4). Another common SNP, ER22/23EK, resulted in almost complete loss of GR activity (Figure 4), which might be associated with decreased sensitivity of glucocorticoids.

## Discussion

The frequent alterations of AR, ERα, and GR genes are clinically important due to their specific functional differences (Table 1). The estimated overall benefit of the molecularly-informed treatment decision was significantly higher over the uninformed (50). In this study, we developed rapid and accurate reporter systems for profiling SHR alterations, which may help individualized treatment decision-making. Our reporter systems overcome the limitations of previously developed dual luciferase reporter assays where several different vectors need to be co-transfected into cells. The inclusion of the *Renilla* reporter gene downstream SHR mRNA under the control of IRES for expression normalization ensures that the firefly luciferase reporter activity accurately reflects the transcriptional activity of SHRs. Our reporter systems also provide a platform for direct assessing and comparing different SHR mutants and variants in their transcriptional activity, which allows for the linking of specific SHR alterations with clinical phenotypes and treatment outcome. Furthermore, our reporter systems are versatile and can be adapted to report the activity of other hormone receptors such as mineralocorticoid receptor (MR), progesterone receptor (PR), and thyroid hormone receptor (THR), by replacing the hormone responsive promoter in the pcDNA3.1(-)-4×ARE-Fluc-AR-Rluc vector with mineralocorticoid receptor elements (MREs), progesterone receptor elements (PREs), or thyroid hormone receptor elements (THRs), and replacing the AR gene with MR, PR, or THR.

Our sensitive assay systems have revealed functional alterations in AR, GR or ERα caused by genetic mutations or splicing variations, with many results being consistent with previous studies (6, 9, 31, 32, 43). However, there are also discrepancies between our results and previous reports. For example, in our study ERα variants including TADDI, ERα36, and ERα46 displayed higher transactivation ability compared with ERα-wt in the presence of DES (Figure 3), but these variants displayed distinct transcriptional activities in other cell lines (51, 52). These discrepancies could result from the different cell lines used which may express different levels of co-regulators of SHRs. Some ERα mutants including E380Q, S463P, L469V, L536H, L536R, Y537C, and Y537D were reported to have an modest increase in transactivation activity in the absence of estradiol (E2), compared to ERα-wt (19, 34, 53). In our study, although some of these mutants showed the same trend as previous studies, the changes in transcriptional activity are not statistically significant (Figure 3). We hypothesized that some variants or mutations may be more effective than ERα-wt in ligand independent activation, but they are not the main causes of disease progression. In addition to transcriptional activity, mutations or splicing variations in SHRs may also cause changes in their half-life and sensitivity to promiscuous agonists, and crosstalk with other factors. Future work is needed to further improve reporter systems for SHRs in order to better inform disease mechanisms and treatment.

## Data Availability Statement

The datasets generated for this study are available on request to the corresponding author.

## Author Contributions

FX, ML, and LW conceived the study and designed the experiments. HJ, YL, MT, ZL, and LZ performed the experiments. FS, YZ, JZ, and HL made clinical validation. LL, BA, and JM collected clinical interpterion. HJ and FX wrote the manuscript. All authors read and approved the final manuscript.

### Conflict of Interest

The authors declare that the research was conducted in the absence of any commercial or financial relationships that could be construed as a potential conflict of interest.
