# Supplementary material for: Quantitative Evaluation of the Transcriptional Activity of Steroid Hormone Receptor Mutants and Variants Using a Single Vector With Two Reporters and a Receptor Expression Cassette
Source: Front Endocrinol (Lausanne). 2020 Mar 31;11:167. doi: 10.3389/fendo.2020.00167 (PMC7137763; doi:10.3389/fendo.2020.00167)
Supplement: Supplementary file 1 [file Data_Sheet_1.docx]

Supplementary Materials for

**Quantitative Evaluation of the Transcriptional Activity of Steroid Hormone Receptor Mutants and Variants Using a Single Vector with two Reporters and a Receptor Expression Cassette**

Authors: Huimin Ji^1,2,3^, Ying Li^3,4^, Zhao Liu^5^, Min Tang^6^, Lihui Zou^4^, Fei Su^7^, Yaqun Zhang^8^ Junhua Zhang^4^, Hexin Li^6^, Lin Li^5^, Bin Ai^5^, Jie Ma^9,11^, Lunan Wang^1,2,3#^, Ming Liu^8#^, Fei Xiao^4,7,10#^

^1^National Center for Clinical Laboratories, ^4^The Key Laboratory of Geriatrics, ^6^Department of Oncology, ^7^Clinical Biobank, ^8^Department of Urology, ^9^Center for Biotherapy, ^10^Department of Pathology, Beijing Hospital, National Center of Gerontology, Chinese Academy of Medical Sciences, Beijing, P. R. China.

^2^Beijing Engineering Research Center of Laboratory Medicine, Beijing Hospital, Beijing, P. R. China

^3^Graduate School, Peking Union Medical College, Chinese Academy of Medical Sciences, Beijing, P. R. China

^5^Department of Thyroid and Breast Surgery, The Affiliated Hospital of Xuzhou Medical University, Xuzhou, P. R. China.

^11^State Key Lab of Molecular Oncology, National Cancer Center, Chinese Academy of Medical Sciences and Peking Union Medical College, Beijing , P. R. China.

^#^**Correspondence should be addressed to:** F.X. ([xiaofei3965@bjhmoh.cn](mailto:xiaofei3965@bjhmoh.cn)) or M. L. ([liuming3222@bjhmoh.cn](mailto:liuming3222@bjhmoh.cn)) or ([lnwang@nccl.org.cn](mailto:lnwang@nccl.org.cn))

Supplementary Text

Fig. S1. Comparison of androgen regulation of the PSA61-luc promoter in Hela, Huh-7, HEK-293T, HepG2, COS-7 and PC3 Cell lines. Firefly luciferase levels were normalized using corresponding *Renilla* luciferase levels for each condition. The results were calculated as the fold stimulation of given plasmids treated with 10 nM R1881 compared to untreated cells transfected with the same plasmids. Data shown are mean ± SD of triplicate.


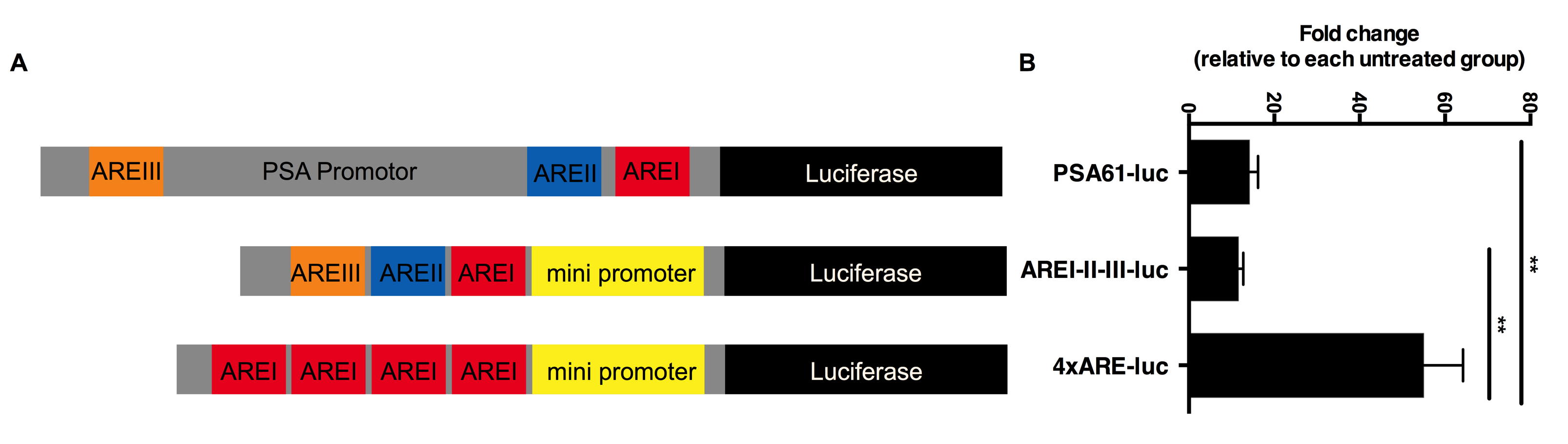


Fig. S2. Stimulation ability of PSA61-Luc, ARE-I-II-III-Luc and 4xARE-Luc

**A**. Schematic representation of the structures of three firefly luciferase reporter plasmids. **B.** Comparison of firefly luciferase reporter plasmids. Firefly luciferase activity of PSA61-Luc, ARE-I-II-III-Luc and 4xARE-Luc in HEK293T cells treated with 10 nM of R1881 or vehicle for 48 hours. Values are means of a minimum of three independent experiments performed in quadruplicate ± SD.. *, *P* < 0.05, **, *P* < 0.01.


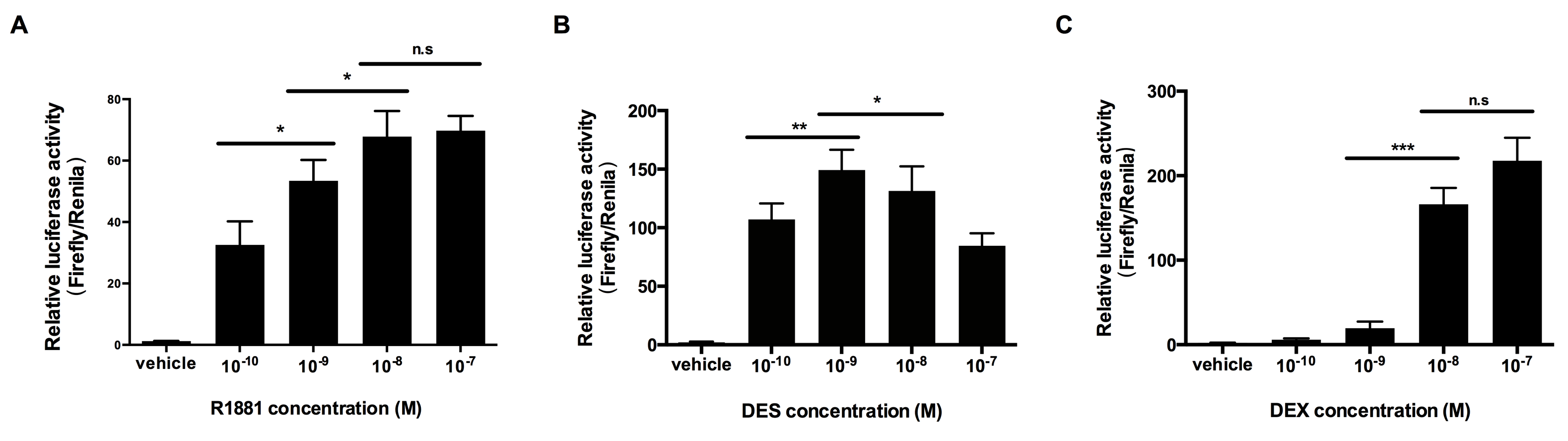


**Fig. S3. Stimulation abilities of 4×SRE-Luc in HEK293T cell line at different conditions .**

**A.** Stimulation ability of 4×ARE-Luc in HEK293T cell line treated with different concentrations (10^-7^, 10^-8^, 10^-9^, 10^-10^ M) of R1881 or vehicle for 48 hours. **B.** Stimulation ability of 4×GRE promoter in HEK293T cell line treated with different concentrations (10^-7^, 10^-8^, 10^-9^, 10^-10^ M) of DES or vehicle for 48 hours. **C**. Stimulation ability of 4×ERE promoter in HEK293T cell line treated with different concentrations (10^-7^, 10^-8^, 10^-9^, 10^-10^ M) of DEX or vehicle for 48 hours. Values are means of a minimum of three independent experiments performed in quadruplicate ± SD. DES: Diethylstilbestrol; DEX: Dexamethasone. *, *P* < 0.05, **, *P* < 0.01, n.s, no significant difference.

Table S1.

| **AR-wt and Mutation** | **oligonucleotide (5’-3')** |
| --- | --- |
| AR-wt-F | gtaggtggaagattcagccaagctcaag |
| AR-wt-R | ctgggtgtggaaatagatgggcttg |
| AR-M523V-F | gtcaaaagcgaaGtgggcccatggatgg |
| AR-M523V-R | ccatccatgggcccaCttcgcttttgac |
| AR-T575A-F | gtcactatggagctctcGcatgtggaagctgcaag |
| AR-T575A-R | cttgcagcttccacatgCgagagctccatagtgac |
| AR-R629Q-F | ggatgactctgggagctcAgaagctgaagaaacttgg |
| AR-R629Q-R | ccaagtttcttcagcttcTgagctcccagagtcatcc |
| AR-L701H-F | tcctttgcagccttgcActctagcctcaatgaa |
| AR-L701H-R | ttcattgaggctagagTgcaaggctgcaaagga |
| AR-W742C-F | cattcagtactcctgTatggggctcatggtgt |
| AR-W742C-R | acaccatgagccccatAcaggagtactgaatg |
| AR-W742L-F | tcattcagtactcctTgatggggctcatggtg |
| AR-W742L-R | caccatgagccccatcAaggagtactgaatga |
| AR-H874Y-F | ttgcgagagagctgTatcagttcacttttgacc |
| AR-H874Y-R | ggtcaaaagtgaactgatAcagctctctcgcaa |
| AR-F876L-F | gagctgcatcagttAacttttgacctgctaat |
| AR-F876L-R | attagcaggtcaaaagtTaactgatgcagctc |
| AR-T877A-F | gagagagctgcatcagttcGcttttgacctgctaatcaagtc |
| AR-T877A-R | gacttgattagcaggtcaaaagCgaactgatgcagctctctc |

Primer sets used to amplify AR-wt and AR mutations

Table S2.

| **ERα-wt and Mutations** | **Oligonucleotide(5’-3')** |
| --- | --- |
| ERα-wt-F | atgaccctccacaccaaa |
| ERα-wt-R | ctcgagtcagaccgtggcagggaaac |
| ERα-A350F-F | gggcttactgaccaacctgTTCgacagggagctggttcaca |
| ERα-A350F-R | tgtgaaccagctccctgtcGAAcaggttggtcagtaagccc |
| ERα-E380Q-F | atgatcaggtccaccttctaCaatgtgcctggctagagat |
| ERα-E380Q-R | atctctagccaggcacattGtagaaggtggacctgatcat |
| ERα-S463P-F | tggagtgtacacatttctgCCcagcaccctgaagtctctg |
| ERα-S463P-R | cagagacttcagggtgctgGGcagaaatgtgtacactcca |
| ERα-L469V-F | tgtccagcaccctgaagtctGtggaagagaaggaccatat |
| ERα-L469V-R | atatggtccttctcttccaCagacttcagggtgctggaca |
| ERα-P535H-F | agtgcaagaacgtggtgAccctctatgacctgctgct |
| ERα-P535H-R | agcagcaggtcatagagggTcaccacgttcttgcact |
| ERα-L536P-F | tgcaagaacgtggtgccccCctatgacctgctgctggag |
| ERα-L536P-R | ctccagcagcaggtcatagGggggcaccacgttcttgca |
| ERα-L536R-F | tgcaagaacgtggtgccccGctatgacctgctgctgggag |
| ERα-L536R-R | ctccagcagcaggtcatagCggggcaccacgttcttgca |
| ERα-L536Q-F | tgcaagaacgtggtgccccAGtatgacctgctgctggag |
| ERα-L536Q-R | ctccagcagcaggtcataCTggggcaccacgttcttgca |
| ERα-L536H-F | tgcaagaacgtggtgccccActatgacctgctgctggag |
| ERα-L536H-R | ctccagcagcaggtcatagTggggcaccacgttcttgca |
| ERα-Y537A-F | caagaacgtggtgcccctcGCtgacctgctgctggagat |
| ERα-Y537A-R | atctccagcagcaggtcaGCgaggggcaccacgttcttg |
| ERα-Y537E-F | caagaacgtggtgcccctcGaAgacctgctgctggagat |
| ERα-Y537E-R | atctccagcagcaggtcTtCgaggggcaccacgttcttg |
| ERα-Y537S-F | caagaacgtggtgcccctctCtgacctgctgctggagat |
| ERα-Y537S-R | atctccagcagcaggtcaGagaggggcaccacgttcttg |
| ERα-Y537C-F | caagaacgtggtgcccctctGtgacctgctgctggagat |
| ERα-Y537C-R | atctccagcagcaggtcaCagaggggcaccacgttcttg |
| ERα-Y537D-F | caagaacgtggtgcccctcGatgacctgctgctggagat |
| ERα-Y537D-R | atctccagcagcaggtcatCgaggggcaccacgttcttg |
| ERα-Y537N-F | caagaacgtggtgcccctcAatgacctgctgctggagat |
| ERα-Y537N-R | atctccagcagcaggtcatTgaggggcaccacgttcttg |
| ERα-Y537K-F | caagaacgtggtgcccctcAaGgacctgctgctggagat |
| ERα-Y537K-R | atctccagcagcaggtcCtTgaggggcaccacgttcttg |
| ERα-D538G-F | aacgtggtgcccctctatgGcctgctgctggagatgct |
| ERα-D538G-R | agcatctccagcagcaggCcatagaggggcaccacgtt |
| ERα-D538N-F | aacgtggtgcccctctatAacctgctgctggagatgct |
| ERα-D538N-R | agcatctccagcagcaggtTatagaggggcaccacgtt |
| ERα-Y537N/D538G-F | aacgtggtgcccctcAatgGcctgctgctgagcatctc |
| ERα-Y537N/D538G-R | agcatctccagcagcaggCcatTgaggggcaccacgtt |
| ERα-Y537S/D538G-F | F: aacgtggtgcccctctCtgGcctgctgctgagcatctc |
| ERα-Y537S/D538G-R | R: agcatctccagcagcaggCcaGagaggggcaccacgtt |

Primer sets used to amplify ERα-wt and ERα mutations

Table S3.

| **GR-wt and Mutation** | **oligonucleotide (5’-3')** |
| --- | --- |
| GR-wt-F | ccgctcgagatggactccaaagaa |
| GR-wt-R | gctctagatcacttttgatgaaacag |
| GR-N363S- F | cccgttggttccgaaaGttggaataggtgc |
| GR-N363S-R | gcacctattccaaCtttcggaaccaacggg |
| GR-D641V-F | cctgcatgtacgTccaatgtaaacacatgc |
| GR-D641V- R | gcatgtgtttacattggAcgtacatgcagg |
| GR-V729I- F | tctatgcatgaagtgAttgaaaatctcctt |
| GR-V729I-R | aaggagattttcaaTcacttcatgcataga |
| GR-V571A- F | ggagggcggcaagCgattgcagcagtgaaa |
| GR-V571A-R | tttcactgctgcaatcGcttgccgccctcc |
| GR-I747M- F | aagaccatgagtatGgaattccccgagatg |
| GR-I747M-R | catctcggggaattcCatactcatggtctt |
| GR-V423A- F | aaactctgcctggCgtgctctgatgaagct |
| GR-V423A-R | agcttcatcagagcacGccaggcagagttt |
| GR-L672P- F | aaaccttactgcttcCctcttcagttccta |
| GR-L672P-R | taggaactgaagagGgaagcagtaaggttt |
| GR-R714Q- F | cagaactggcagcAgttttatcaactgaca |
| GR-R714Q-R | tgtcagttgataaaacTgctgccagttctg |

Primer sets used to amplify GR-wt and GR mutations

Table S4.

| **AR variants** | **Oligonucleotide(5’-3')** |
| --- | --- |
| AV-V1-R1 | caaacaccctcaagattctttcagaaacaacaacagctgctcccagagtcatccctg |
| AV-V1-R2 | ttaaggaagccattctgagactccaaacaccctcaagatt |
| ARV2-R1 | ctgctggcgcacaggtacttctgtttccctcccagagtcatccctgcttcataacattt |
| ARV2-R2 | ttcggaatttatcaatagtgcaatcatttctgctggcgcacaggtacttctgtttccct |
| ARV3-R1 | ttagtgtctgatgattcttttaatttgttcattctgaaaaatccttcagcggctctttt |
| ARV3-R2 | ttctgtcagtcccattggtgctgccatgcagtatggcttggggttagtgtctgatgatt |
| ARV3-R3 | ttcaagtttgtttctctgtaggatttcttcctatttctgttgttttctgtcagtcccat |
| ARV3-R4 | cctaagtatgattcaaaggccatgagacagctttcaagtttgtttct |
| AR-V4-R | ctatgattcttttaatttgttcattctgaaaaatcctcccagagtcatcc |
| AR-V5-R | tcagtctcccagagtcatccct |
| AR-V6-R | ctatgacactctgctgcctgctcccagagtcatccctgc |
| AV-V7-R1 | tgagatgcttgcaattgccaacccggaatttttctcccagagtcatccctgc |
| AV-V7-R2 | ccgctcgagtcagggtctggtcattttgagatgcttgcaatt |
| AR-V8-R | ttaactagataattcacagaggttgtcaaaacctcccagagtcatccc |
| AR-V9-R1 | gcaaatgtctccaaaaagcagcttgctcaggtaagttgtctcccagagtcatccc |
| AR-V9-R2: | ttacttcttaacaacgtgatcccaaaagatgtgcaaatgtctccaaa |
| AR-V10-R1 | atccctgtgaggtaggaaaacactattggtcccgctggagggagtcatccctgcttca |
| AR-V10-R2 | atgaatggtaaccactattacttctacagcccgtcctcacaacatccctgtgaggtagg |
| AR-V10-R3 | ttacaggaaacaataatcatgatactcacaactacatgaatggtaaccac |
| AR-V11-R1 | aggaggaggaagagaaagaaaagtatcttacctcccagagtcatccct |
| AR-V11-R2 | ttagaaaatgagggagaagggggagagaggaaggaggaggaagaga |
| AR-V12-R | tcaaaagtgaactgatgcagctctctcgcaatcaggcaaggccttggccc |
| AR-V13-R | tcaggtgtgattaatgctgaagagtag |
| AR-V14-R | tcacaagtacatggcatcaggtgtgataggctgcacggagtc |
| AR-V15-R1 | atcatgtcctctactctttttatccctctgctccttcacaggactaatgctgaagagta |
| AR-V15-R2 | tcatctgtcttgaactagtaaagtacaatgtatcatgtcctctact |
| AR-V15-R3 | ttagttccattgagtttttatgctttccacattcatctgtcttgaac |
| AR-V16-R | tcacaagtacatggcatcaggtgtgatcatgtgtgacttgat |
| AR-V17-R1 | acaaaactgcatcctctctcacaagtacatggcatcaggtgtgaccagaaaggatcttg |
| AR-V17-R2 | tgttctgagagagcttccaaaacaaaactgcatcctctct |
| AR-V17-R3 | ttaatccaggtgtcttgtttgttctgagagagctt |
| AR-V18-R1 | gcatcctctctcacaagtacatggcatcaggtgtgatctggaactaatgctgaagagta |
| AR-V18-R2 | ctgagagagcttccaaaacaaaactgcatcctctctcaca |
| AR-V18-R3 | ttaatccaggtgtcttgtttgttctgagagagcttcca |
| AR23-R | agacctgataatgagtttccagagtctctttcttcgggtatttcttcagcggctctttt |
| AR23-F | gaaactcattatcaggtctatcaactcttgtatttgttctcccagggaaacagaagtac |
| AR45-F | atgatactctggcttcacagtttggagactgccagg |
| AR8-R1 | gatagacctgataatgagtttccagagtctctttcttcgggtatttcgcatgtccccgt |
| AR8-R2 | tctgtttccctgggagaacaaatacaagagttgatagacctga |
| AR8-R3 | tcatttctgctggcgcacaggtacttctgtttccctggga |
| AR-Q640X-R | ctatagtttcagattacca |

Primer sets used to amplify AR variants

Table S5.

| **ERα variants** | **Oligonucleotide(5’-3')** |
| --- | --- |
| TIDDI-R | cattggtactggccaatctttcatcattcccacttcgtagcattt |
| TIDDI-F | aaagattggccagtaccaatgttgaaacacaagcgccagagagat |
| ERa36-F | atggctatggaatctgccaa |
| ERa36-R1 | aggatgcaagttcaggattctcttctttgcttctacatgtgagataccagaattaagca |
| ERa36-R2 | tcagacacgaggaaaccacttgtttccaaatattttaggatgcaagttca |
| ERa46-F | atggctatggaatctgccaaggagactcg |
| ER-V2-R | ttatgtcctgtagaatgccggcgggccggc |
| ER-V3-F | agtattcaagggatacgaaaagaccgaagaggagggagaa |
| ER-V3-R | tttcgtatcccttgaatacttctcttgaagaaggccttgc |
| ER-V4-F | gaaaggtggctttgtggatttgaccctccatgatcag |
| ER-V4-R | ccacaaagccacctttcatcattcccacttcgtagc |
| ER-V5-R | ctacacattttccctggttcctggcaccctcttcgcccagt |
| ER-V6-F | cttggacaggagtgtacacatttctgtccagcaccc |
| ER-V6-R | gtacactcctgtccaagagcaagttaggagcaaac |
| ER-V7-R1 | cctttgttacagaattaagcaaaataata |
| ER-V7-R2 | tcatgctgtacagatgctccatgcctttgttacagaat |

Primer sets used to amplify ERα variants

Table S6.

| GR variants | Oligonucleotide(5’-3') |
| --- | --- |
| GRβ-R1 | tgtgctttctggttttaaccacataacattttcatgcatagaatc |
| GRβ-R2 | gctctagatcagattaatgtgtgagatgtgctttctggttt |
| GR-P-R | gctctagactaccaacctgaagagagaagcag |
| GRγ(insAGG)-F | gagcagtggaaggaaggcagcacaattacc |
| GRγ(insAGG)-R | ggtaattgtgctgccttccttccactgctc |
| GRγ(insGTA)-F | gagcagtggaaggagtacagcacaattacc |
| GRγ(insGTA)-R | ggtaattgtgctgtactccttccactgctc |
| GR-A-F | ggaatgaacctggaagttcctaaggacggt |
| GR-A-R | accgtccttaggaacttccaggttcattcc |
| GR-B –F | ccgctcgagatggacttctataaa |
| GR-C1-F | ccgctcgagatgggactgtatatg |
| GR-C2-F | ccgctcgagatgggagagacagaa |
| GR-C3-F | ccgctcgagatgggaaatgacctg |
| GR-D1-F | ccgctcgagatgtctgccatttct |
| GR-D2-F | ccgctcgagatgtaccactatgac |
| GR-D3-F | ccgctcgagatgaatacagcatcc |
| GR-ER22/EK23-F1 | ggtagagaagaaaaccccagcagtgtgcttgctcaggagaagggagatgtga |
| GR-ER22/EK23-F2 | ggtagagaagaaaaccccagcagtgtgcttgctcaggagaagggagatgtga |

Primer sets used to amplify GR variants

Table S7.

| **Name** | **Oligonucleotide(5’-3')** |
| --- | --- |
| ARE | AGAACAGCAAGTGCT |
| ERE | TGTACAGGATGTTCT |
| GRE | GTCAGGTCACAGTGACCTGAT |

Sequences of ARE, ERE and GRE
